# Synthesis of the Humites *n*Mg_2_SiO_4_·Mg(F, OH)_2_

**DOI:** 10.6028/jres.065A.043

**Published:** 1961-10-01

**Authors:** A. Van Valkenburg

## Abstract

The humite group comprises four minerals having the general formula *n*Mg_2_SiO_4_·Mg(F, OH)_2_ where *n* = 1, 2, 3, and 4. These have been synthesized in the laboratory from melts, by solid state reactions, and by hydrothermal techniques. Complete substitution of GeO_2_ for SiO_2_ has been accomplished in three of the humites and partial substitution of OH ions for F, Fe for Mg, and Ti for Mg or possibly Si, has been accomplished in one or more of the series. Indices of refraction of the synthetic humites are given and powder X-ray diffraction data have been obtained for the fluorine end members. Also, infrared data from 4,000 to 300 cm^−1^ are given for the fluorine end members and these are compared to the corresponding spectra of the natural analogs.

## 1. Introduction

The term “humites” as used by mineralogists refers to four minerals that have the general formula *n*Mg_2_SiO_4_·Mg(F, OH)_2_, where *n*−1, 2, 3, or 4. The individual mineral names are norbergite, *n* = 1, named from the Norberg locality, Vasmanland, Sweden; chondrodite, *n* = 2, named from the Greek word meaning “a grain,” alluding to the granular texture of the material as it occurs in aggregate form; humite, *n* = 3, named after Sir Abraham Hume; and clinohumite, *n* = 4.

The synthesis of the humites was undertaken as part of a general study to determine the factors governing the isomorphous substitution of fluorine for hydroxyl groups in hydrous silicate structures. The natural humites contain both fluorine and hydroxyl anions, but fluorine is always present as the major constituent. This fact suggests that a complete isomorphous substitution of hydroxyl for fluorine may not be possible in the humites. The hydrothermal studies of Bowen and Tuttle [1]^1^, Roy and Roy [2], and Carlson et al., [3] in the system SiO_2_–MgO–H_2_O failed to produce a hydrous analog of the humites and their results support the idea that a complete isomorphous substitution of fluorine by hydroxyl cannot take place.

The primary objectives of the humite synthesis were to (1) determine the minimum ratio of F^−1^ to (OH)^−1^ that can exist in a given structure; (2) determine some of the properties of end members; and (3) determine the possible existence of humite members having *n* = 5, 6, etc., or ½ ¼, etc.

Daubree in 1851 [4] and Dolter [5] in 1889 may have synthesized one or more of the humites by reacting fluorides with silicates in the absence of water, although their identifications were not conclusive. W. Jander and V. R. Fett [6] in 1939 made a series of hydrothermal syntheses in which they claimed to have synthesized chondrodite and humite, using formula proportions of oxides and HF. Their data on pressure and temperature relationships are lacking and they apparently placed no significance on the presence of extra lines or absence of lines in the X-ray powder diffraction data of their synthetic materials. During World War II, German scientists [7] reported the synthesis of the humites as impurities in the flakes of synthetic fluorophlogopite. The presence of the humites in the mica was attributed to an incorrect composition of the mica melt. In 1947 [8] Rankama synthesized norbergite and chondrodite by fusing forsterite with magnesium fluoride in a graphite crucible. His attempts to produce hypothetical members of the group where *n* = 5, 6, etc., were not successful.

## 2. Synthesis Techniques

The humites were synthesized at the National Bureau of Standards using the techniques of crystallization from melts, hydrothermal reactions, and solid state reactions. The fluorohumites, containing all fluorine and no hydroxyl groups, were synthesized using solid state reactions and crystallization from melts. The hydroxyl-bearing humites were synthesized under hydrothermal conditions. The starting materials consisted of reagent grade magnesium carbonate, magnesium fluoride and silicic acid or quartz, In special cases natural forsterite (Mg_2_SiO_4_) and olivine (Mg,Fe)_2_SiO_4_ were used with MgF_2_ to give the desired formula proportions. The raw batches were prepared by first sintering together magnesium carbonate and silica to drive off CO_2_ and water vapor before adding MgF_2_. This practice was undertaken to prevent the MgF_2_ from prematurely decomposing, releasing F_2_ at temperatures below fusion. After sintering, MgF_2_ was added in formula proportions to the batch and the ingredients were reground in a mechanical mortar to insure thorough mixing. To obtain maximum reactivity of a batch using solid state reaction techniques, the powder was compressed into ½-in. diam wafers at a pressure of 20,000 psi. The wafers were then placed in platinum envelopes which were sealed by crimping adjacent edges. Another technique employed was to tamp powder into a platinum cylinder of ½ mm ID by ½ cm long and hermetically seal both ends. Platinum resistance furnaces with controllers were used to heat and melt the specimens. Temperatures were measured in degrees C to a precision of ±3° using Pt-Pt 10 percent Rh thermocouples. The thermocouples were placed on the outside wall of the platinum envelopes and cylinders.

## 3. Thermal Dissociation Characteristics of the Synthetic Fluorohumites

**Figure 1:**
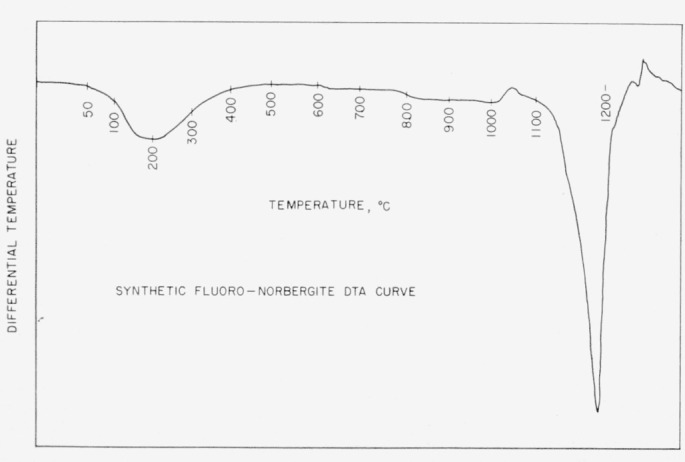
Synthetic fluoronorbergite DTA heating curve.

The dissociation temperature of norbergite was the only dissociation temperature determined in the humites as the experimental conditions were not satisfactory for the other humites. Two techniques were used to determine the norbergite dissociation temperature. The first employed the well-known soak-quench technique developed by the Geophysical Laboratory, and the second employed a differential thermal apparatus. Initially experiments were designed to quench materials in a platinum envelope that was not sealed. It was found that volatiles escaped from the envelope changing the composition, thus giving erroneous results. Sealed platinum envelopes were then used and these gave reproducible results. Unfortunately, the dissociation temperatures of the remaining humites were too high for the sealed envelopes and they burst at temperatures above 1,300 °C. The dissociation temperature of the fluoronorbergite, using the quench technique, was 1,180±5 °C. A differential thermal analysis curve, figure 1, obtained from a sealed platinum envelope indicates dissociation at approximately 1,195 °C, as observed in the endothermic peak at this temperature. The small exothermic peak at 1,050 °C is unexplained and the broad endothermic peak at 200 °C is due to the elimination of hygroscopic water. On cooling an exothermic peak occurred at 1,180 °C, indicating recrystallization. This point is not recorded on the heating curve in figure 1. The differential curve was made on a conventional apparatus employing a heating rate of 12 °C/min.

Evidence from sealed tube experiments indicated that norbergite melts incongruently. At temperatures above 1,180 °C, a norbergite composition yielded chondrodite as the major phase.

## 4. Synthesis of the Fluorohumites by Solid State Reactions

The fluorohumites were readily synthesized using solid state reaction techniques. The advantages of using these techniques are that the series can be crystallized at temperatures lower than their fusion temperatures and losses of volatile constituents can be kept at a minimum. The reactions yielding the best results were those in which the powdered ingredients were compressed into pellets and then enclosed in platinum tubes. Norbergite was readily synthesized by reacting at temperatures of 1,150 °C for periods of several hours. The remaining humites were synthesized at higher temperatures in the range of 1,250 to 1,300 °C. Hot pressing methods were also employed in which wafers 1 in. in diameter and ¼ in. thick were pressed and heated in a carbon resistance furnace at 1,400 psi and 1,100 to 1,300 °C.

Considerable difficulty was experienced in identifying the individual synthetic humites from microscopic examinations until it was realized that the synthetic analogs of chondrodite and clinohumite may exhibit apparent orthorhombic symmetry. Natural chondrodite and clinohumite are monoclinic and in chondrodite the extinction angle of *X*∧*a* = 26 to 30°, while in clinohumite *X*∧*a* = 9°±. As in the reaction products of the chondrodite and clinohumite compositions straight extinction was observed, it was assumed that these compounds were not synthesized. However, the disturbing feature was the presence of phases that had indices of refraction equivalent to a fluorochondrodite and fluoroclinohumite. This problem was discussed with H. F. W. Taylor, who has synthesized a hydroxyl calcium analog of chondrodite [10]. He has concluded from calculations that a chondrodite structure can exhibit orthorhombic symmetry providing certain conditions are met. These conditions are based on the stacking arrangements of the alternating layers of Mg_2_SiO_4_ and MgF_2_ found in the humites [11], and the layering is governed by repeated twinning on a unit cell scale.

Table 1 lists the refractive indices of the synthetic fluorohumites as measured by oil immersion techniques and white light. These indices and all others given in this paper are accurate to ±.002. The value of 2*V* is estimated.

Since the compositions of the humites depend on the alternate layering of Mg_2_SiO_4_ and Mg(F,OH)_2_ it was thought that compositions corresponding to 5Mg_2_SiO_4_·MgF_2_ or ½ Mg_2_SiO_4_·MgF_2_ might be synthesized. Stoichiometric compositions of these were prepared and heated at various temperatures. An examination of the rection products did not indicate the formation of other humite compounds.

## 5. Infrared Spectra

The infrared spectra of the synthetic fluorohumites are presented to show differences in the spectra of individual compounds that can be used in diagnostic identifications. The spectra were made on a double-beam spectrophotometer using a NaCl prism in the region of 4,000 to 900 cm^−1^ and a CsBr prism in the region 800 to 300 cm^−1^. The samples were prepared by grinding in a boron carbide mortar, then mixing the powder with dry ground KBr and firmly pressing the ingredients into pellets at 100,000 psi using standard techniques. The sample concentration ranged from 2 to 3 mg per gram of KBr.

In figures 2a to 5b are plotted infrared spectrograms of the synthetic fluoro and natural humites between 4,000 and 300 cm^−1^. The major absorption bands of these compounds are located in the region between 1,050 and 850 cm^−1^ and they are believed to be caused by the stretching modes of the Si–O bond. The specific assignments of individual bands were not attempted in these studies. The natural norbergite samples came from Franklin, N.J., and contained adhered impurities of calcite in estimated amounts of 5 percent or less by volume. The natural chondrodite sample was also from Franklin, N.J., and it contained some calcite impurities that were estimated to be less than 5 percent by volume. The natural humite and clinohumite samples were from Monte Somma, Italy, and these were quite pure, with impurities estimated to be less than 0.5 percent by volume. [Fig f2b-jresv65an5p415_a1b] [Fig f3a-jresv65an5p415_a1b] [Fig f3b-jresv65an5p415_a1b] [Fig f4a-jresv65an5p415_a1b] [Fig f4b-jresv65an5p415_a1b] [Fig f5a-jresv65an5p415_a1b]

### 5.1. Norbergite Spectra

The infrared spectra of the natural and synthetic norbergites show differences. The band at 3,500 cm^−1^ in the synthetic material is due to O–H stretching of water in the KBr pellet, while in the same band of the natural material the O–H stretching is due to hydroxyl groups in the norbergite. These hydroxyl groups can be distinguished from the water found in KBr by their greater intensity when compared to a test KBr pellet. In the natural norbergite, the bands located at 1,460 cm^−1^ and 880 cm^−1^ are caused by calcium carbonate impurities. The band at 760 cm^−1^ in the natural sample is absent in the synthetic sample. The band occurring at 625 cm^−1^ in the natural sample has shifted to 630 cm^−1^ in the synthetic sample: also, the band at 565 cm^−1^ in the natural sample has shifted to 570 cm^−1^ in the synthetic material.

### 5.2. Chondrodite Spectra

The synthetic chondrodite spectra show two major differences as compared to the natural chondrodite. The band at 760 cm^−1^ in the natural sample is entirely absent in the synthetic. A similar absence is noted in the synthetic material for the band at 960 cm^−1^. The sharp peak at 3,500 cm^−1^ indicates the presence of OH in the structure of the natural chondrodite. The bands at 618 cm^−1^ and 555 cm^−1^ in the natural material have shifted to 625 cm^−1^ and 560 cm^−1^ in the synthetic material. The band at 768 cm^−1^ in the natural material is absent in the synthetic sample.

### 5.3. Humite Spectra

The humite patterns are essentially the same except for the differences in the OH bands at 3,500 cm^−1^. The sharper band in the natural humite indicates hydroxyl groups. The band at 755 cm^−1^ in the natural sample is absent from the synthetic sample and the band at 560 cm^−1^ in the synthetic is missing or obscured in the natural pattern. In the pattern of the synthetic sample there are bands at 493, 420, and 365 cm^−1^ which may be obscured or missing in the patterns of the natural material. Again a shift is noted from band 615 cm^−1^ of the natural material to 620 cm^−1^ in the synthetic sample.

### 5.4. Clinohumite Spectra

The clinohumite spectra show differences in the intensities of the 1,000 cm^−1^ and 890 cm^−1^ bands. In the synthetic sample, the band at 890 cm^−1^ is more intense than the 1,000 cm^−1^ band. The reverse is true for the natural clinohumite. The pattern of the natural mineral has a band at 725 cm^−1^ that is absent for the synthetic sample. The band at 610 cm^−1^ for the mineral is displaced to 620 cm^−1^ in the pattern of the synthetic humite. The band at 540 cm^−1^ in the synthetic-sample pattern is obscured by the shoulder of the mineral pattern. Bands found at 425, 390, and 370 cm^−1^ for the synthetic humite are not identified in the pattern of the mineral.

The differences between the patterns of the synthetic and natural humites are not explained as no assignments of the absorption bands were attempted. The synthetic fluorohumites differ chemically from the natural analogs in that no hydroxyls are present in their structures. The natural samples all contain hydroxyls in addition to fluorine. Also, the analyzed natural samples show the presence of FeO, Fe_2_O_3_, Al_2_O_3_, and TiO_2_ and these oxides may perturb the bonds between atoms sufficiently to account for some shifting of the bands. It is also known that the synthetic chondrodite and clinohumite show apparent orthorhombic symmetry as compared to the monoclinic symmetry of the natural analogs. These differences should also affect the absorption patterns.

X-ray powder diffraction patterns were made of the synthetic fluorohumites using an X-ray diffractometer with Cu K*_α_* radiation, (1.5405 A) at 25° C. The samples used for X-ray analysis were estimated under the microscope to contain 1 percent or less impurities by volume. A spectrographic analysis showed the following impurities; less than 1.0 percent iron; 0.1 percent each of aluminum and calcium; and less than 0.01 percent each of boron, chromium, manganese, nickel, strontium, and titanium. The norbergite and humite samples have been indexed and the results are given in tables 2 and 4. In tables 3 and 5 the *d*-spacings for chondrodite and clinohumite are given. For purposes of comparison a norbergite examined by the British Museum, ASTM card number 2–1345, and a norbergite reported by Sahama [12] have been included in table 2. Also, natural chondrodite, humite, and clinohumite, as reported by Sahama [12], have been included in tables 3, 4, and 5. It should be noted that the natural humites contain hydroxyl units as well as fluorine.

Taylor and West [11] in 1929 determined that norbergite has the space group Pnma (No. 62) and 4(Mg_2_SiO_4_·MgF_2_) per unit cell. Table 2a gives a comparison of the lattice constants of Taylor and West’s norbergite with the NBS synthetic fluoronorbergite.

The humite structure as determined by Taylor [11] and West has a space group Pnma (No. 62) and 4(3Mg_2_SiO_4_·MgF_2_) per unit cell. Table 4a gives a comparison of the lattice constants of Taylor and West’s humite with NBS synthetic fluorohumite.

## 6. Hydrothermal Synthesis of the Humites

The two major objectives in the hydrothermal synthesis of the humites were to determine (1) the maximum amount of hydroxyl ions (OH)^−1^ that can replace fluorine ions F^−1^ in the structure of a given humite and (2) the hydrothermal stability range of the series.

The apparatus used in the hydrothermal experiments is the same as that developed at the Geophysical Laboratory [9]. The ingredients used in all experiments consisted of MgO, SiO_2_ as precipitated silica and MgF_2_ as the fluorinating agent. The ingredients were ground in a mechanical mortar to ensure thorough mixing and to increase the grain surface area for better reactivity. Charges weighing about 0.2 g and distilled water were hermetically sealed in platinum tubing ½ in. long by ⅛ in. O.D. The platinum tubing was then placed in the hydrothermal apparatus. The distilled water was always added to the charge in excess of that required for stoichiometric proportions.

The reaction products obtained from the hydrothermal experiments were usually fine grained and it was difficult to identify the phases microscopically although an occasional grain was observed that was large enough to obtain optical properties. Consequently, identification of fine-grained phases was made using X-ray powder diffraction data. However, it was thought unreliable to determine by this means whether hydroxyl ions were substituting for fluorine ions in any given experiment. Past experiences have indicated there is very little structural change in the unit cell, and certainly there is very little change in X-ray scattering power, when substitution occurs. Thus there is little or no change in the X-ray patterns.

However, owing to the relatively large differences in optical refractivities of hydroxyl compounds relative to isomorphous fluorine analogs, refractive index measurements are likely to yield values of the extent of replacement, if use is made of Gladstone and Dale’s principle [13] stating

$$K=\frac{n-1}{d}.$$

*d* is the density which can be assumed constant for OH/F replacements in isomorphous silicates owing to the similarity of radius and mass of the ions; the average refractive index is $n=\sqrt[{3}]{\alpha \beta \gamma }$, and *K* is the specific refraction, a simple additive function:

$$K=\Sigma m_{i}K_{i}.$$

The *m_i_*’s are the mass fractions of constituents of specific refraction *K_i_*. Experience shows [13] that the values of *K* are most reliable when they are computed from a closely similar compound of known composition and specific refraction *K* = *K*_0_. For the humite group one can compute *K*_0_ for the fluorinated compounds and then write

$$K=K_{0}+Zm_{A}[K_{\text{Mg}{\left(\text{OH}\right)}_{2}}-K_{{\text{MgF}}_{2}}].$$

*Z* is the desired fraction of F^−^ groups replaced by (OH)^−^; *m_A_* is the average of the mass fractions of Mg(OH)_2_ and MgF_2_ in the hydroxylated and fluorinated formulas, respectively. Using known values for refractive indices and densities of sellaite and brucite $K_{\text{Mg}{\left(\text{OH}\right)}_{2}}-K_{{\text{MgF}}_{2}}=0.114$. Thus,

$$Z=\frac{K-K_{0}}{0.114m_{A}}=\frac{\partial n}{d\cdot 0.114m_{A}}.$$

+ ∂*n* is the difference in average refractive index observed between the compound of unknown composition and the fluorinated analog. The calculation can be readily followed from table 5a.

A brief consideration of the sources of error in the calculation of the values of *Z* will show readily that the mathematical simplifications in the above analysis introduce an error of less than 1 percent. Similarly the density values are better than would correspond to a ½ percent accuracy, and the assumption of density constancy with composition over the limited range is unlikely to affect *Z* by more than 1 percent. Uncontrolled compositional variations in the synthetic humites are not only small, but are known to affect the refractive index but narrowly.

A further possible source of error could arise from Gladstone and Dale’s principle not applying within the accuracies here considered. From experience [15] one can be confident that for the limited replacements here discussed the application of the principle is sound.

It remains only to discuss the experimental uncertainties introduced from the refractive index measurements. The commonly claimed third decimal figure is rightly suspected owing to some systematic errors which, however, are largely eliminated when only a difference in refractive indices is involved as in this experiment. The 0.002/∂*n* values given in table 52 are the author’s estimate of the fractional errors which account for the largest uncertainties in the determinations of *Z*. An approximate estimate of the probable error in *Z* is obtained by multiplying 0.002 *∂n* by *Z* (see table 5a).

## 7. Norbergite Compositions

Fourteen hydrothermal experiments were completed on the norbergite composition as shown in table 6. The first seven experiments were made using a calculated F^−1^ to (OH)^−1^ ratio of 1:1. These ratios and the subsequent ratios were obtained in the raw batch composition by varying the proportions of MgF_2_ to Mg(OH)_2_. The next six experiments were run on compositions that were calculated to yield a completely fluorinated norbergite. In experiment 14 fluorine was eliminated from the composition with the objective of synthesizing norbergite having only hydroxyl groups.

The norbergite compositions containing a calculated ratio of 1:1, F^−1^ to (OH)^−1^ indicate that chondrodite forms as the stable phase at 700 to 800 °C and 20,000 psi. At 700 °C crystallization is inhibited and the presence of some chondrodite and talc indicates that this temperature may be near the lower stability limit of chondrodite formation. At 600 °C the presence of norbergite was determined by X-ray analysis. Below 600 °C talc appears to be the stable phase and this talc may contain fluorine in its structure. These hydroxyl bearing chondrodites also showed orthorhombic symmetry under the microscope, as zero extinction angles were observed parallel to the major crystallographic axes. The chondrodite obtained from the norbergite composition of experiment 1 contained a few grains that were large enough to measure indices of refraction: *α* = 1.600, *β* = 1.611, and *γ* = 1.628. These indices are higher than the indices measured on a completely fluorinated chondrodite grown from solid state reactions where *α* = 1.582, *β* = 1.594, and *γ* = 1.612. The higher indices indicate the presence of hydroxyl groups in the structure. Calculations further show that 26 percent of the F^−1^ ions have been replaced by (OH)^−1^ ions (see table 5a).

Experiments 8 to 13 were made using a norbergite composition calculated to give an anhydrous fluoronorbergite. Since the ingredients were exposed to water vapor there was always the possibility of (OH)^−1^ entering the structure in preference to F^−1^. In these experiments norbergite formed in the temperature range of 500 to 800 °C and a pressure range of 16,000 to 20,000 psi. In experiment 9 a few grains of norbergite were large enough to measure indices of refraction: *α* = 1.560, *β* = 1.564, and *γ* =1.580. In comparison the indices of an anhydrous synthetic norbergite made from solid state reactions are *α* =1.548, *β* =1.552, and *γ* = 1.570. Again the higher indices of refraction indicate that hydroxyl units are present in the norbergite structure and that 10 percent of the F^−1^ have been replaced by hydroxyls (see table 5a). It is significant to note here that the composition was calculated to give an anhydrous norbergite. In the reaction, however, (OH)^−1^ ions were incorporated in the structure and there was no optical evidence of any variation in the indices of refraction indicating that the OH content was the same for all the grains examined. This suggests that the maximum amount of (OH)^−1^ has entered the structure as none of the experiments showed norbergite grains with higher indices of refraction.

In these norbergite experiments it is to be noted that the amount of fluorine present may alter the course of reaction. For example, in experiment 1, where the ratio of F^−1^ to (OH)^−1^ is 1:1, the major phase as determined by X-ray powder data was chondrodite. In comparison, experiment 9 was calculated to have 100 percent F and the pressure, temperature and time are essentially the same. The major phase, however, was norbergite.

Experiment 14 was one of several attempts to synthesize a hydroxyl norbergite but none of these was successful. The reaction products were the same as those found by Bowen and Tuttle, namely, talc, brucite, forsterite, etc. [1]. Fluorine appears to be a necessary constituent in norbergite.

## 8. Chondrodite Compositions

Chondrodite compositions were prepared in the same manner as the norbergite compositions and a list of experimental results are given in table 7. Experiments 1, 2, and 3 contained a calculated 1:1 ratio of F^−1^ to (OH)^−1^. Chondrodite formed in all three experiments and, in addition, brucite was present in the reaction products formed at 500 °C and 20,000 psi (experiment 3), while humite formed at 800 °C and 20,000 psi (experiment 1). The humite of this experiment had indices of *α* = 1.620, *β* = 1.629 and *γ* = 1.650. In comparison the anhydrous humite containing fluorine gave *α* = 1.598, *β* = 1.606, and *γ* =1.630. It would appear that hydroxyl groups had also entered the humite structure. The hydroxyl content in humite was calculated to be 45 percent (see table 5a). Experiments 4, 5, and 6 contained a calculated 100 percent F in (F, OH) and their reaction products contained chondrodite. Experiment 7 did not contain any fluorine and it was made under the same conditions as experiment 6 with the idea of synthesizing a hydroxyl chondrodite. Forsterite was the major constituent in this experiment and there was no evidence of any chondrodite.

## 9. Humite Compositions

Table 8 lists the results of experiments on humite compositions. The major phases obtained in these experiments are either forsterite or talc, and humite did not appear as a reaction product. It should be noted again that water was added to the humite composition in excess of that required for the hydroxyl content, and this excess water may well be responsible for decreasing the fluorine ion concentration thus shifting the course of reaction away from humite.

## 10. Clinohumite Compositions

Compositions corresponding to clinohumite were prepared and run under the same conditions as the humite experiments. The results of these experiments were the same as the humite experiments, and there was no optical evidence that a clinohumite containing hydroxyl units was synthesized. A table of the clinohumite compositions was not prepared.

## 11. Substitutions

It is well known that germanium will proxy for silicon atoms in many of the silicate structures. Robbins and Levin [13] have succeeded in synthesizing a germanium forsterite, Mg_2_GeO_4_. Since the forsterite structure is one of the fundamental building blocks in the humites, it was thought that germanium humites could be synthesized. Using solid state reaction techniques, germanium analogs of silicon fluorohumites were synthesized with the exception of clinohumite. This analog was not identified in any of the reaction products. However, the search was not exhaustive and it is thought likely that the compound can be synthesized provided the correct condition of temperature, composition, etc., is found. As with the other synthetic fluorochondrodites, the germanium fluorochondrodite shows orthorhombic symmetry under the polarizing microscope. The germanium analogs are positive in optical character with the following indices as determined by oil immersion techniques and white light.

**Table:** 

	*α*	*β*	γ
			
Germanium norbergite	1.590	1.598	1.638
Germanium chondrodite	1.658	1.670	1.735
Germanium humite	1.716	1.732	1.758

Buckle and Taylor [10] were successful in obtaining a calcium analog of chondrodite, Ca_5_(SiO_4_)_2_(OH)_2_, using hydrothermal techniques. The other three members of the humite group were not reported but it seems reasonable that, given the proper conditions, they also can be synthesized. One of the important features of this synthesis is that a hydroxyl humite without fluorine can be obtained. The author repeated the synthesis of Buckle and Taylor and confirmed their results. Attempts by the author, however, to substitute fluorine for hydroxyl in the calcium analog were not successful.

As titanium has been reported in several analyses of the natural humites [12], a few preliminary experiments were made to introduce TiO_2_ synthetically into the norbergite structure. Two fluoronorbergite compositions were formulated to replace approximately 10 and 20 percent of MgO by TiO_2_. The two compositions were fired at 1,150 °C in one experiment and at 1,250 °C in another. For comparison a TiO_2_ free norbergite was run at the same time for each experiment. In all the TiO_2_ experiments the reaction products yielded a norbergite that had higher indices of refraction than that of the control norbergite. The *α* index of refraction for the TiO_2_ norbergite, and this includes both the 10 and 20 percent compositions, was 1.557 as compared to 1.546 for the control norbergite and the *γ* index was 1.578 as compared to 1.570. No attempt was made to substitute TiO_2_ in the other humite structures, but it is probable that TiO_2_ will go into these structures.

Ferrous iron is found in most natural humites substituting for magnesium and two experiments were designed to incorporate ferrous iron in the synthetic fluorochondrodite structure. This structure was chosen to observe the possible effect of iron on the crystal symmetry of chondrodite. Since previous attempts to synthesize a monoclinic form were unsuccessful it was thought the presence of the slightly larger ferrous ion might change the layering or stacking characteristics of the structure, producing monoclinic symmetry similar to that of the natural analog. A fluorochondrodite composition was prepared using a natural olivine (MgFe)_2_SiO_4_ that contained approximately 15 percent of ferrous iron. To this MgF_2_ was added in formula proportions and the batch heated to 1,380 °C with one sample exposed to atmospheric conditions and a second sample exposed to the inert gas helium to prevent oxidation of the iron. In both experiments a reaction product was obtained that appeared optically to be orthorhombic. There was no evidence of monoclinic symmetry. The reaction products of both experiments had the same indices of refraction with *α* = 1.612 and *γ* =1.642 and their X-ray diffraction patterns were the same and typical of synthetic chondrodites.

## 12. Summary

The humites have been synthesized using the techniques of solid state reactions, hydrothermal reactions, and crystallization from melts. The synthetic fluoro analogs of chondrodite and clinohumite did not have the monoclinic symmetry characteristic of the natural minerals; in comparison they showed orthorhombic symmetry under the microscope. This anomaly is interpreted as a difference in structural stacking in which layered segments are arranged to give either monoclinic or orthorhombic symmetry. X-ray, optical, and infrared data are given for the synthetic fluorohumites and they are compared to those of their natural analogs. The substitutions of germanium for silicon, calcium for magnesium, titanium for magnesium or possibly silicon, ferrous iron for magnesium, and hydroxyls for fluorine were successfully achieved in one or more of the humite series. Experimental evidence indicates that the humite series does not extend beyond compositions corresponding to norbergite or clinohumite.

## Figures and Tables

**Figure 2a:**
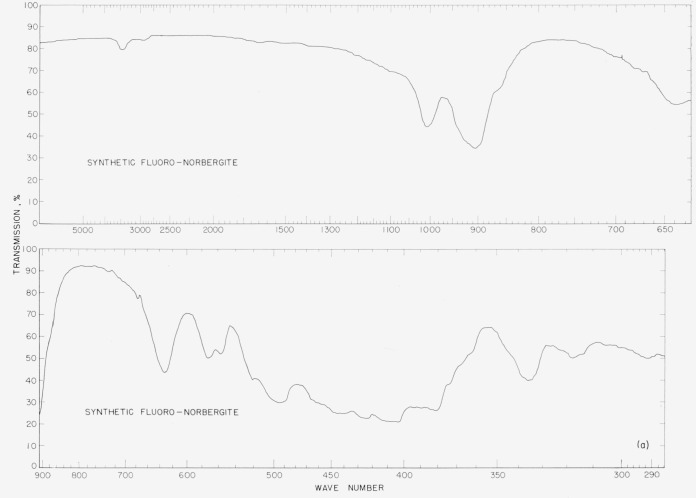
Infrared absorption spectra of synthetic fluoro norbergite.

**Figure 2b f2b-jresv65an5p415_a1b:**
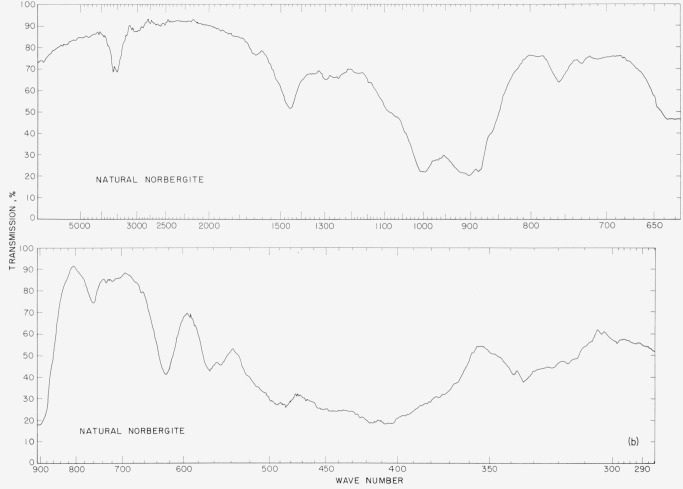
Infrared absorption spectra of natural norbergite.

**Figure 3a f3a-jresv65an5p415_a1b:**
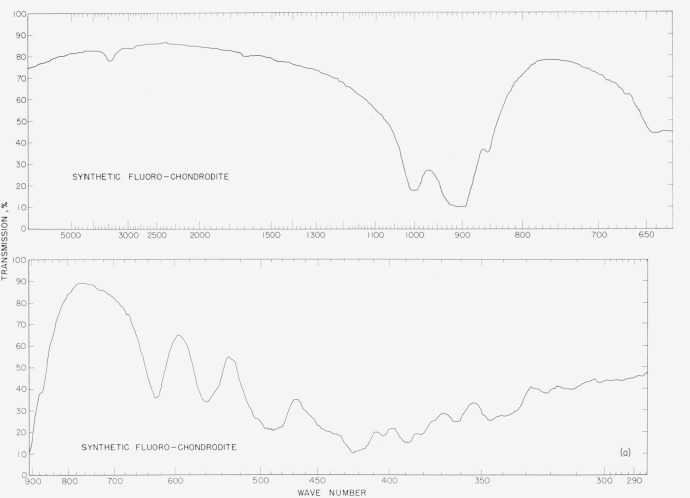
Infrared absorption spectra of synthetic fluoro chondrodite.

**Figure 3b f3b-jresv65an5p415_a1b:**
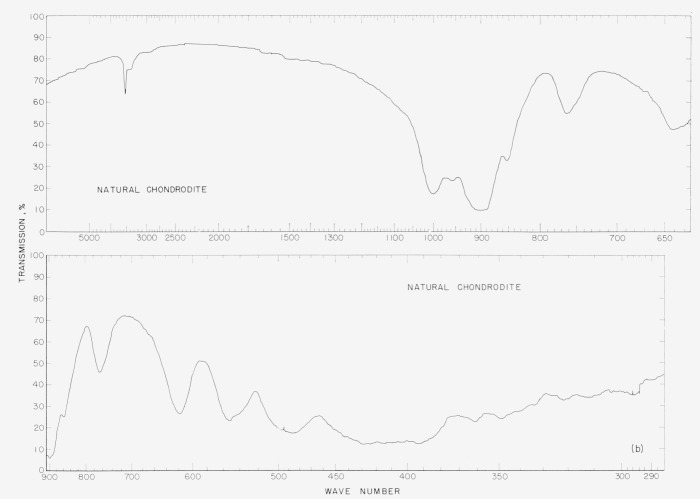
Infrared absorption spectra of natural chondrodite.

**Figure 4a f4a-jresv65an5p415_a1b:**
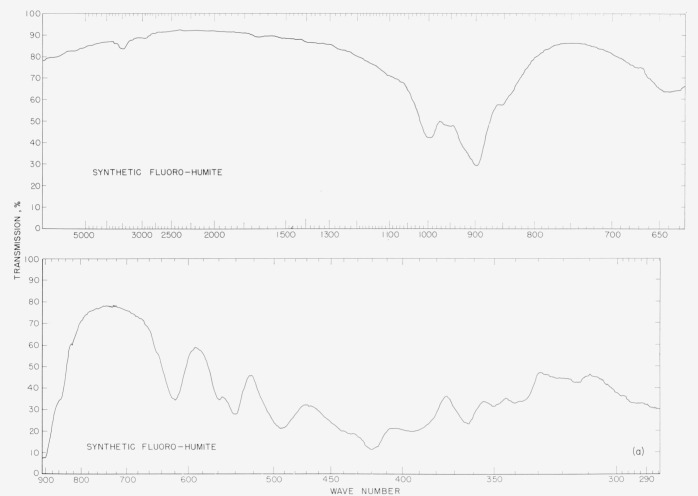
Infrared absorption spectra of synthetic fluorο humite.

**Figure 4b f4b-jresv65an5p415_a1b:**
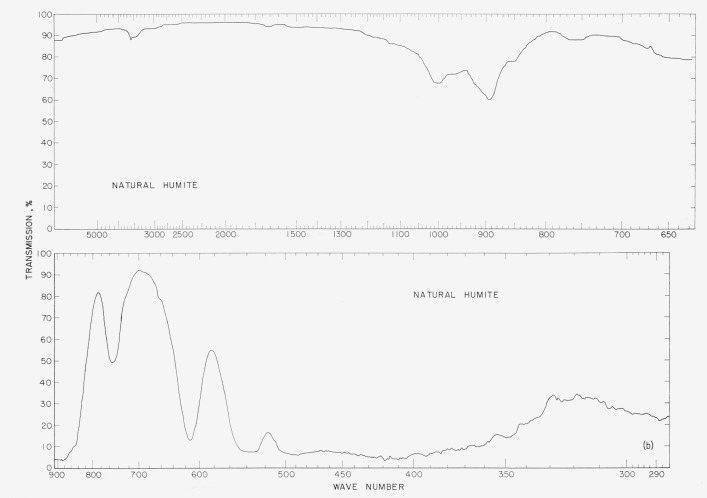
Infrared absorption spectra of natural humite.

**Figure 5a f5a-jresv65an5p415_a1b:**
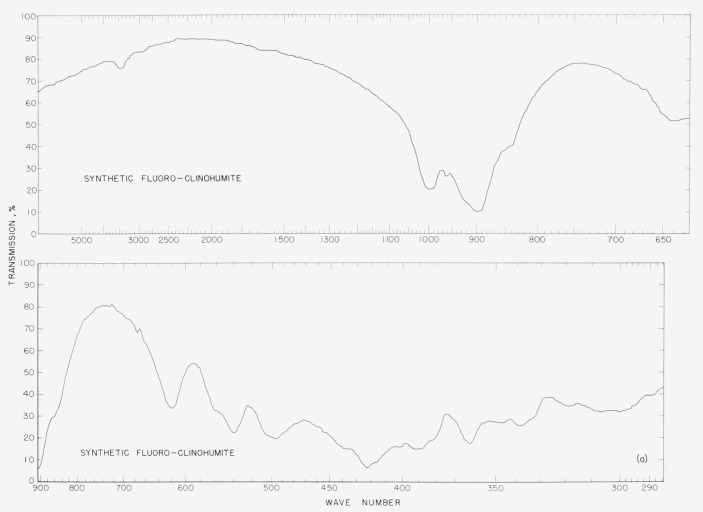
Infrared absorption spectra of synthetic fluoro clinohumite.

**Figure 5b:**
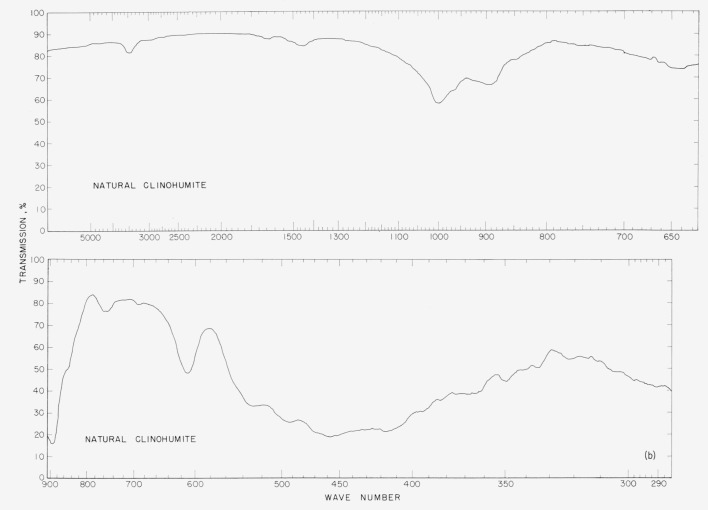
Infrared absorption spectra of natural clinohumite.

**Table 1:** 

	*α*	*β*	*γ*	2*V*
				
Norbergite	1.548	1.552	1.570	33°
Chondrodite	1.582	1.594	1.612	50°
Humite	1.598	1.606	1.630	60°
Clinohumite	1.608	1.618	1.636	75°

**Table 2:** Norbergite

*hkl*	NBS synthetic fluoro Cu, 1.5405 A at 25 °C		British Museum Natural Cu, 1.5418 A		Sahama Natural Cu, 1.5405 A	
	*d*	*I*	*d*	*I*	*d*	*I*
						
	*A*		*A*		*A*	
200	5.145	18	5.2	40	5.13	m
...............	...............	...............	4.8	40	...............	...............
210	4.428	10	4.41	50	...............	...............
020	4.371	28	...............	...............	4.38	w
101	4.283	12	...............	...............	4.27	vw
011	4.149	20	...............	...............	4.14	vw
220	3.327	22	3.37	60	3.32	m
211	3.227	27	...............	...............	3.22	m
121	3.058	100	3.08	80	3.06	vs
...............	...............	...............	2.94	40	...............	...............
301	2.771	14	2.77	40	2.76	vw
221	2.716	12	...............	...............	2.72	vw
311	2.639	73	2.66	70	2.63	s
410	2.466	15	2.52	60	2.46	w
131	2.408	36	2.43	40	2.41	m
321	2.337	34	2.35	40	2.34	m
102	2.296	16	...............	...............	2.29	w
401	2.255	68	2.26	80	2.25	m
231	2.230	80	...............	...............	2.23	s
112	2.214	8	...............	...............	...............	...............
411	2.184	8	...............	...............	...............	...............
122	2.0320	10	2.03	20	2.03	vw
240, 331	2.0008	5	...............	...............	...............	...............
141	1.9442	14	1.94	60	1.948	vw
430	1.9243	6	...............	...............	...............	...............
222	1.9201	5	...............	...............	...............	...............
241	1.8472	8	1.86	20	...............	...............
511	1.8408	9	1.81	20	1.838	vw
322	1.7733	4	...............	...............	...............	...............
402	1.7357	32	1.74	100	1.733	m
232	1.7241	48	1.74	20	1.723	s
341	1.7125	5	...............	...............	1.721	m
412	1.7022	14	...............	...............	1.699	vw
250	1.6529	10	1.65	50	...............	...............
151	1.6160	7	1.60	...............	...............	...............
332, 422	1.6125	10	...............	...............	...............	...............
042	1.6004	9	...............	...............	...............	...............
620	1.5938	10	...............	...............	1.592	vw
611, 142	1.5808	6	...............	...............	...............	...............
441	1.5678	4	...............	...............	...............	...............
103	1.5518	2	...............	...............	...............	...............
502, 013	1.5450	2	1.54	20	...............	...............
242, 113	1.5280	6	...............	...............	...............	...............
432	1.4890	16	...............	...............	1.480	w
213, 351	1.4763	45	1.49	80	1.475	m
123	1.4621	6	1.47	40	1.471	w
522	1.4582	6	...............	...............	1.460	w
060	1.4547	20	...............	...............	...............	...............
450	1.4435	6	...............	...............	...............	...............
301	1.4265	4	...............	...............	...............	...............
313, 631	1.4080	7	1.41	40	...............	...............
701, 260	1.3990	6	(a)	...............	1.398	vw

a Eight additional lines were omitted.

**Table 2a:** Lattice constants

		*a*	*b*	*c*
				
		*A*	*A*	*A*
1929	Taylor and West [12] natural	10.2	8.74	4.71
1959	NBS synthetic fluoro	10.271	8.727	4.709 at 25 °C

The density of norbergite calculated from the NBS lattice constants is 3.194 g/cm^3^ at 25 °C.

**Table 3:** Chondrodite

NBS synthetic fluoro Cu, 1.5405 A at 25 °C		Sahama from Hangelby Natural Cu, 1.5405 A	
*d*	*I*	*d*	*I*
			
*A*		*A*	
7.418	10	...............	...............
4.854	40	4.83	s
3.992	20	...............	...............
3.948	18	...............	...............
3.897	11	3.92	w
3.685	23	3.69	w
3.554	42	3.55	m
3.477	30	3.47	m
3.390	27	3.37	m
3.007	45	3.00	s
2.910	15	2.91	vw
2.842	15	2.83	w
2.764	35	2.75	m
2.672	43	2.66	s
2.605	57	2.61	m
2.512	41	2.50	s
2.461	11	...............	...............
2.428	18	2.42	w
2.318	27	2.31	m
2.301	21	2.29	w
2.272	100	2.27	m
2.252	100	2.25	vs
2.149	16	2.14	vw
2.025	9	2.10	vw
2.009	10	...............	...............
1.945	9	...............	...............
1.882	8	...............	...............
1.851	9	...............	...............
1.795	16	1.790	vw
1.789	16	...............	...............
1.737	98	1.735	s
1.693	16	1.688	vw
1.633	5	...............	...............
1.617	18	1.611	w
1.603	11	...............	...............
1.574	12	1.570	vw
1.561	5	...............	...............
1.542	6	...............	...............
...............	...............	1.498	vw
...............	...............	1.478	m
...............	...............	1.395	vw
...............	...............	1.340	m

**Table 4:** Humite

*hkl*	NBS synthetic fluoro Cu, 1.5405 A at 25*°* C.		Sahama Natural Cu, 1.5405 A	
	*d*	*I*	*d*	*I*
				
	*A*		*A*	
020	10.42	5	..........	..........
042	5.17	9	..........	..........
200	5.11	9	..........	..........
210	4.97	18	4.96	w
220	4.59	19	4.57	w
111	4.20	1	..........	..........
121	3.97	5	..........	..........
031	3.90	12	3.90	vw
131	3.66	5	3.65	m
240	3.34	49	..........	..........
201	3.47	5	3.46	vw
060	3.453	32	..........	..........
211	3.430	25	3.42	w
141	3.312	32	3.31	m
051	3.119	7	3.12	vw
023	3.102	7	..........	..........
151	2.980	6	2.98	vw
241	2.885	6	2.89	vw
301	2.770	23	2.76	w
311	2.744	32	2.74	m
161	2.691	50	2.69	m
321	2.674	5	..........	..........
080	2.589	7	..........	..........
331	2.572	38	2.57	m
420	2.490	4	..........	..........
261	2.453	4	..........	..........
341	2.443	30	..........	..........
171	2.438	70	2.44	s
430	2.399	21	2.40	w
022	2.308	8	..........	..........
351	2.304	12	2.30	vw
440	2.297	7	..........	..........
271	2.256	100	2.26	vs
401, 122	2.251	35	..........	..........
181	2.218	17	..........	..........
132	2.189	7	..........	..........
361	2.158	6	..........	..........
142	2.107	38	..........	..........
222	2.103	30	..........	..........
460	2.057	6	..........	..........
2·10·0,291	1.920	5	..........	..........
501	1.881	7	..........	..........
1·10·1	1.867	8	..........	..........
521	1.850	1	..........	..........
531	1.814	6	..........	..........
471	1.794	2	..........	..........
2·10·1	1.779	6	..........	..........
391	1.772	5	..........	..........
082	1.7477	6	..........	..........
272	1.7387	65	1.740	s
182	1.7229	5	..........	..........
490, 551	1.7117	4	..........	..........
481	1.6995	3	..........	..........
432	1.6859	12	1.685	vw
3·10·1,630	1.6581	10	..........	..........
442	1.6486	1	..........	..........
372	1.6256	2	..........	..........
640	1.6213	15	1.621	w
452	1.6032	4	..........	..........
621	1.5863	5	..........	..........
3·11·1	1.5575	4	..........	..........
462	1.5525	13	..........	..........
1·10·2,123	1.5419	3	..........	..........
2·13·0	1.5223	10	..........	..........
..........	*1.5192	15	1.520	vw
4·11·0	1.5176	5	..........	..........
1·13·1	1.4948	1	..........	..........
..........	*1.4895	8	1.485	m
670	1.4786	69	1.478	w
3·12·1	1.4644	1	..........	..........
1·11·2	1.4588	1	..........	..........
303	1.4226	1	..........	..........
163,2·14·0	1.4223	4	..........	..........
..........	..........	..........	1.396	vw
..........	..........	..........	1.392	vw
..........	..........	..........	1.380	vw
..........	..........	..........	1.344	w

* These “d” spacings cannot be indexed, but since they occur in Sahama’s natural material, they have been included for comparison.

**Table 4a:** Latttice constants

		*a*	*b*	*c*
				
1929	Taylor and West, natural material.	10.23	20.86	4.738
1960	NBS, synthetic material	10.243	20,72	4.735 at 25 °C

The density of humite calculated from NBS lattice constants is 3.201 g/cm^3^ at 25 °C.

**Table 5:** Clinohumite

NBS synthetic fluoro Cu, 1.5405 A at 25 °C		Sahama from Hameenkyla Natural Cu, 1.5405 A	
*d*	*I*	*d*	*I*
			
*A*		*A*	
5.029	25	5.02	s
4.453	22	4.44	w
3.870	29	3.86	w
3.696	47	3.70	s
3.487	30	3.48	w
3.450	30	3.44	w
3.338	16	3.35	vw
3.224	21	3.22	m
2.919	9	2.91	vw
2.768	58	2.76	s
2.675	7	2.68	vw
2.604	27	2.60	w
2.538	59	2.54	s
2.512	59	2.51	s
2.459	19	..........	..........
2.405	22	2.40	w
2.391	16	2.39	vw
2.354	32	2.36	m
2.312	14	2.30	vw
2.257	100	2.26	s
2.218	7	..........	..........
2.188	9	2.15	vw
2.156	15	..........	..........
1.879	10	..........	..........
1.826	5	..........	..........
1.796	8	..........	..........
1.790	6	..........	..........
1.768	8	..........	..........
1.741	80	1.742	s
..........	..........	1.738	vs
1.683	14	1.681	vw
1.656	4	..........	..........
1.636	7	..........	..........
1.624	17	1.624	vw
1.609	16	1.612	vw
1.541	8	1.537	vw
..........	..........	1.479	m
..........	..........	1.396	vw
..........	..........	1.345	w

**Table 5a:** 

Name	*n* Fluorinated	*n* Unknown composition containing hydroxyls	*∂n*	$\frac{0.002}{\partial n}$	*d*	*m_A_*	*Z* for unknown composition
							
					*g/cm* ^3^		%
Norbergite	1.557	1.568	0.011	0.2	3.19	0.314	10±2
Chondrodite	1.569	1.613	.017	.1	3.12	.179	26±3
Humite	1.612	1.633	.021	.1	3.12	.130	45±5
Clinohumite	...............	...............	...............	...............	...............	...............	...............

**Table 6:** Norbergite compositions hydrothermal experiments

Experiment	%F in (F,OH)	Temp. °C	Psi	Time	Results
					
1	50	810	20,000	1 week	Major phase as determined by X-ray Chondrodite *α* = 1.600, *β* = 1.611, *γ* = 1.628.
2	50	800	20,000	21 hr	Same as above.
3	50	800	8,000	16 hr	Chondrodite major phase.
4	50	700	20,000	16 hr	Some brucite. Chondrodite is beginning to crystallize.
5	50	700	8,000	5 days	Chondrodite. Material not well crystallized.
6	50	600	20,000	1 week	Material not well crystallized. Major phase norbergite as determined by X-ray.
7	50	400	16,000	1 week	Under microscope, reaction appeared very poor. X-rays indicated the presence of some talc.
8	100	810	16,000	1 week	Norbergite plus small amount of talc.
9	100	700	20,000	3 days	Norbergite major phase, *α* = 1.560, *β* = 1.564, *γ* =1.580.
10	100	700	20,000	16 hr	Norbergite.
11	100	600	20,000	1 week	Norbergite major phase.
12	100	500	16,000	1 week	Norbergite major phase.
13	100	400	20,000	3 days	Talc major phase.
14	0	700	18,000	5 days	Forsterite, periclase with some brucite.

**Table 7:** Chondrodite compositions hydrothermal experiments

Experiment	%F in (F,OH)	Temp.	Psi	Time	Results
					
		*°C*			
1	50	800	20,000	16 hr	Crystals of humite present. *α* = 1.620, *γ* = 1.650 Major phase chondrodite.
2	50	700	8,000	2 weeks	Chondrodite.
3	50	500	20,000	3 days	Major phases brucite and chondrodite.
4	100 (F)	800	20,000	16hr	Chondrodite and talc. No humite.
5	100 (F)	700	8,000	2 weeks	Chondrodite and talc.
6	100 (F)	500	20,000	3 days	Talc, brucite, and chondrodite.
7	0	700	10,000	2 weeks	X-ray pattern close to that of forsterite.

**Table 8:** Humite compositions hydrothermal experiments

Experiment	%F in (F,OH)	Temp.	Psi	Time	Results
					
		*°C*			
1	50	900	20,000	3 days	Major phase forsterite.
2	50	800	20,000	3 days	Small leak developed. Forsterite, talc and possible humite.
3	50	600	20, 000	3 days	Major phase is talc.
4	50	700	8,000	5 days	Forsterite and some talc.
5	100	900	20,000	3 days	Forsterite and second phase not identified.
6	100	800	20,000	3 days	Forsterite major phase.
7	100	700	8,000	5 days	Forsterite major phase.
8	100	600	20,000	3 days	Talc major phase.
9	0	700	10,000	5 days	Forsterite and talc major phase.

## Footnotes

1 Figures in brackets indicate the literature references at the end of this paper.
